# Seemingly unrelated time series model for forecasting the peak and short-term electricity demand: Evidence from the Kalman filtered Monte Carlo method

**DOI:** 10.1016/j.heliyon.2023.e18821

**Published:** 2023-08-09

**Authors:** Frank Kofi Owusu, Peter Amoako-Yirenkyi, Nana Kena Frempong, Akoto Yaw Omari-Sasu, Isaac Adjei Mensah, Henry Martin, Adu Sakyi

**Affiliations:** a National Institute for Mathematical Sciences (NIMS), Faculty of Physical and Computation Science, College of Science, KNUST-Kumasi, Ghana; b Department of Mathematics, Faculty of Physical and Computation Science, College of Science, KNUST-Kumasi, Ghana; c Department of Statistics and Actuarial Science, Faculty of Physical and Computation Science, College of Science, KNUST-Kumasi, Ghana; d Institute of Applied Systems Analysis (IASA), School of Mathematics, Jiangsu University, Zhenjiang 2102013, Jiangsu, PR China; e Department of Physics, Faculty of Physical and Computational Science, College of Science, KNUST-Kumasi, Ghana

**Keywords:** Seemingly unrelated times series model, Peak electricity demand, Short-term electricity demand

## Abstract

In this extant paper, a multivariate time series model using the seemingly unrelated times series equation (SUTSE) framework is proposed to forecast the peak and short-term electricity demand using time series data from February 2, 2014, to August 2, 2018. Further the Markov Chain Monte Carlo (MCMC) method, Gibbs Sampler, together with the Kalman Filter were applied to the SUTSE model to simulate the variances to predict the next day's peak and electricity demand. Relying on the study results, the running ergodic mean showed the convergence of the MCMC process. Before forecasting the peak and short-term electricity demand, a week's prediction from the 28th to the 2nd of August of 2018 was analyzed and it found that there is a possible decrease in the daily energy over time. Further, the forecast for the next day (August 3, 2018) was about 2187 MW and 44090 MWh for the peak and electricity demands respectively. Finally, the robustness of the SUTSE model was assessed in comparison to the SUTSE model without MCMC. Evidently, SUTSE with the MCMC method had recorded an accuracy of about 96% and 95.8% for Peak demand and daily energy respectively.

## 1 Introduction

Electricity is one of the major determinants of the economic prosperity of any country. It plays a significant role in undertaking daily activities and very essential for quality healthcare delivery, education, transport, effective communication, mineral exploration, and many more; serving as the building block on which every sector of a nation's economy thrives. This emphasizes how crucial and indispensable electricity is for human existence in the 21st century [1]. A reliable and continuous supply of electrical energy is necessary for the functioning of today's complex societies. There are three major stages in power supply from the power plant to end users of the electricity; namely, generation, transmission, and distribution [2]. Due to the increasing consumption and obstruction of various kinds and the extension of existing electrical transmission networks all across the world, power systems are operating closer to their limits. Therefore, the probability of occurrences of overloading, equipment failures, and blackout tend to increase. Moreover, another problem in this sector is that electrical energy cannot be stored in larger quantities; hence, electricity is only generated when needed [3].

Since electricity operators supply different kinds of distributors and consumers who are noted for different behaviors, there are fluctuations in the demand and supply of electricity. Changes in weather conditions, consumption patterns, population growth, consumer income, and behavior, and electricity prices among others have also contributed to the stochastic nature of electricity production and supply. Specifically, the stochastic power demand from the consumers’ side plays a vital role together with the fluctuating character of solar energy and renewable energy supply. Further, changing consumption patterns can magnify the issues associated with stochastic power generation. For instance, it is likely that in the near future, the transport sector will come to rely on electricity, which will create a degree of exposure to variations in electricity supply that does not exist in the sector at the moment. Another scenario is the increasing tendency of households to generate power via photovoltaic panels or other local general means as well as consuming it from the grid. Such producers or consumers (prosumers) may be less predictable in their consumption patterns than traditional consumers especially since they are more likely to participate in demand management programs that encourage them to dynamically their use of power. The introduction of energy mixing systems within renewable sources of electricity (for example, solar energy) has also increased the complexity of the entire electricity process. This is due to its high unpredictability and unreliable nature [4]. Yet, the priority of every operator is to make the supply as reliable as possible. This goes a long way to affect the electricity market since it influences the mechanisms employed by operators to meet shortfalls or excess of generation and supply at short notice. Furthermore, energy in terms of electricity has to be consumed as soon as it is produced, and power demand together with supply have to remain essentially balanced in real-time for the grid frequency to remain stable [5]. Due to this, forecasting energy demand has become an important component of the energy management system.

Forecasting energy demand, mainly referring to forecasting electricity demand and energy, is being used throughout all segments of the electric power industry, including generation, transmission, distribution, and retail. It is the prediction of electrical power required to meet the short-term, medium-term, or long-term demand. Energy demand forecasts have been playing a vital role in the electric power industry for over a century [5]. Applications of energy demand forecasts spread power supply planning, transmission and distribution systems planning, demand-side management, power systems operations and maintenance, financial planning, rate design, and other smaller units. Energy demand forecasting is a central and integral process in the planning and operation of electric utilities [6].

Overall, electricity storage limitations and the societal necessity of electricity lead to several interesting features of forecasting the demand for energy, such as the complex seasonal patterns, data collection across the grid, and the need to be extremely accurate [5]. Many countries have made some structural changes in their electricity systems; privatizing the power systems, creating electricity markets thereby increasing competition, splitting up monopolies, and promoting greater penetration of renewable generation within the system. The highly unpredictable and unreliable nature of these renewable sources has made the electricity system more complex. Yet energy produced must be reliable and generated in the needed amount for efficient usage. Due to this reason, every electricity operator is particular about the amount of energy to supply to meet the demand of the consumers and to reduce the cost of transmission as well. If the right information about the demand is not given, the distributor is likely to oversupply or undersupply energy to consumers leading to higher production costs of supply. In addition, under-supplying electricity might lead to blackouts and low productivity of consumers. With all these challenges facing the electricity companies, Ghana Grid Company Limited (GRIDCo), which is in charge of the transmission process in Ghana, has no standard model for daily forecasting.

There have been several models developed to address load forecasting issues [7]. The times series model is one of the statistical methods that have been extensively employed. For instance, Shah et al. [8] used the component estimate method to forecast Pakistan's energy usage one month ahead. The power consumption time series was separated into two key components, deterministic and stochastic. Parametric and nonparametric methods were implemented under the deterministic component while the stochastic component methods included Autoregressive (AR), Nonparametric Autoregressive (NPAR), Smooth Transition Autoregressive (STAR), and Autoregressive Moving Average (ARMA). P-ARMA and NP-ARMA produced the best results but the NP-NPAR model stayed competitive with the top. Javed et al. [9] conducted research on short-term electricity forecasting by implementing methods that could take care of the non-linearity induced by the non-linear seasonal data which in the long run affects the demand for electricity. In view of this, Output Error (OE), Auto-Regressive with Exogenous Inputs (ARX), Auto-Regressive Moving Average with Exogenous Inputs (ARMAX), Support Vector Machine (SVM), Artificial Neural Network–Particle Swarm Optimization (ANN–PSO), K-Nearest Neighbor (KNN), Artificial Neural Network–Levenberg–Marquardt algorithm with one and two hidden layers and bootstrap aggregation models were employed for this research with ANN-LM predicting better as compared to each other since it captures non-linearity in data. Luis et al. [10] conducted a study at a luxury 5-star hotel in the South of Tenerife, Spain to predict short-term electricity forecast. In this research, several models were employed which includes an ARIMAX model which performed well by predicting with an accuracy of 94.22%. In addition, to predict energy demand for non-residential buildings, an ARIMA model, a polynomial model, a neural network model, and a support vector machine model were employed [11]. The research was based on data from the University of Deusto in Donostia-San Sebastian, Spain, on energy usage. The kind of day, work versus non-work, and prior demand for energy for the “q” days of the same sort of day as the next projected were utilized as inputs for each model. The MAPE for the ARIMA model of the database employed by the University was 7.34%, 7.92% for the Support Vector Machine model, 11.91% for the Polynomial model, and 13.46% for the Neural Net model. This research revealed that the ARIMA model outperformed the other models with an accuracy of about 93%. Further Katara et al [12] predicted electricity consumption in Tamale, Ghana, using an ARIMA model. The study used secondary data from the Northern Electricity Department Tamale, covering from 1990 to 2013, and categorized electricity usage into domestic, commercial, and industrial. ARIMA (1, 1, 3) was identified as the optimal model for forecasting the demand with about 90% accuracy. To forecast Ghana's electricity consumption by 2030 Sarkodie [13], utilized the Autoregressive Integrated Moving Average (ARIMA) model. Using time series data from 1980 to 2013, the ARIMA projection indicated that Ghana's energy usage would rise from 8.52 billion kWh in 2012 to 9.56 billion kWh in 2030.

There have been substantial efforts to predict the energy demand in terms of electricity and peak demand across the world but not enough work has been done in Ghana [14]. According to the literature, there has been less focus on predicting the peak demand along with the predicted demand for energy. The demand peak highly influences the demand for electricity. If the peak goes beyond expected, it is likely to increase the demand for the day, thereby incurring more costs for operations. This extant paper, therefore, seeks to forecast the daily peak demand and daily total energy to be generated by applying the Seemingly Unrelated Time Series Model (SUTSM). Sub-Saharan Africa's electrical demand is growing due to fast population growth, resulting in increasing demand. Despite a 2.45% drop in the number of people without access to electricity between 2013 and 2018, the electrification rate has remained low at 45% ([13,15] According to Chaaraoui et al. [15], unreliable energy supply affects about 80% of Sub-Saharan African enterprises, owing to outdated power grid technology and inadequate network management. The Ghanaian energy industry suffers from substantial transmission and distribution losses of about 20%, while electricity prices are growing due to inflation and bad contracting with foreign power plant providers, resulting in a transition from low electricity availability to low affordability [16]. Proper planning will therefore be able to reduce the losses in the transmission and distribution processes by avoiding oversupply or undersupply of electricity. This can be done only by forecasting energy and Peak forecasting.

The electrical industry in recent times has been liberalized in most nations. Hence, trading of electricity is done in deregulated electricity markets. Electricity demand in these markets is decided the day before delivery through (semi-)hourly simultaneous auctions. As a result, precise projections are critical for efficient and effective power system management [17]. Electricity demand and pricing, on the other hand, have unique characteristics such as non-constant mean and variance, jumps, calendar impacts, various periodicities, high volatility, and so on, which complicate forecasting [17]. Taking into account the elaborated research discussed, it is evident that numerous studies in the area of electricity demand and pricing have not applied specifically applied the seemingly unrelated time series model. Based on outlined evidence, this study seeks to apply the seemingly unrelated time series equation (SUTSE) model to forecast the peak and short-term electricity demand via the Kalman Filter Monte Carlo approach. Compared to other methods applied in the field for forecasting the peak and electricity demand, the SUTSE model has the ability to predict the next value after the last observation more accurately than subsequent forecast values. This is possible because of the implementation of the Kalman Filter and the Markov Chain Monte Carlo (MCMC) approach which predicts the true states of the measurement by incorporating both the observed and quantifying its associated errors in the prediction. This MCMC method due to its conditional probabilities is able to learn from observations, other parameters, and the previous values of the parameters through a Monte Carlo process. This thus makes it more efficient as compared to other parameter estimation methods since it comes up with several scenarios and the by the law of large numbers, estimates the ergodic means from the process.

Considering the eminence of this extant research, with the idea of reducing losses in the transmission process, forecasting becomes very relevant. Hence models to be adopted should be highly accurate and therefore with minimal errors. The Kalman filter method incorporates the idea of combining the measurements or actual observations together with the uncertainty attached to these observations to estimate their true states and hence reducing the error attached to the output. Moreover, the Markov Chain Monte Carlo (MCMC) has also proven to be a more efficient parameter estimation method as compared to traditional methods. This is due to the application of the Monte Carlo method and the introduction of the dependence structure in the parameter estimation. Further electricity companies mostly face the challenge of overproduction or underproduction of electricity. Most of these companies, especially the transmission, including the Ghana Grid Company are into predicting the next day's total energy to be supplied or transmitted. This is to reduce the probability of oversupply or undersupply and hence reduce the cost of production. These institutions also predict the peak demand since the higher the peak, the higher the probability of an increase in demand. Also, the peak demand prediction, especially a 1-day ahead prediction has become very relevant since at the peak, the lines or the system generates heat due to possible overloading on the network, hence losing energy through heat. Losses also tend to increase the cost of production. This thus practically makes it expedient for this type of study.

Electricity demand forecasting is a critical issue for both energy companies and policymakers, as accurate predictions of future electricity demand can help to optimize resource allocation and ensure the electrical grid's reliability and stability. However, due to the complexity and uncertainty of the underlying factors affecting electricity demand, it can be difficult in terms of forecasting. Using state space models to model the interrelationships between multiple time series is, therefore, one approach to addressing these challenges. State space models capture the dynamic interrelationships between multiple time series by explicitly modeling the underlying latent states. The traditional time series methods which have been applied extensively, even in Ghana fail to capture the model dynamics that occur at these time points. This research thus proposed a more robust model, a Kalman-filtered Monte Carlo State Space model which uses the idea of latent states and the Kalman filter Algorithm to capture hidden processes or the dynamics that occurs at discrete time points in the system.

The rest of the article has been structured to cover the methodology, results, and discussions, together with the conclusion and recommendation for future research sections, respectively.

## 2 Methodology

The necessity for reliable electrical load forecasting ranges from short to long-term. The national system requires a balance between produced and consumed electricity at all times of the day, so short-term forecasting is critical. For the planning of new electrical utilities, long-term forecasting is essential. Forecasting errors have significant financial consequences. The goal of this work is to create a new method for projecting daily electricity loads in the short term. As previously mentioned, numerous studies have been written in recent years about approaches and models for load forecasting. Between statistical models and exponential smoothing approaches, between univariate models and models incorporating explanatory variables, and between linear and nonlinear models, contributions can be differentiated. In previous papers, single-equation models and multiple-equation models with various equations for different hours of the day were constructed. There are independent multiple-equation models and correlated multiple-equation models. Specifically, the time dependence of both daily and hourly loads has been captured in observation-driven ARIMA and VARIMA types models and parameter-driven models with unobserved components. In this extended paper, therefore, we formulate a forecasting model based on an interpretable decomposition of electrical loads in time-varying-seasonal effects.

Notably, our proposed model is inspired by Brons et al. [18] and Havranek et al [19] who performed a meta-analysis to enquire about the variation in empirical estimates of price elasticity of electricity demand through developing an estimation method based on the SUTSE model. Thus, different from the studies conducted by Brons et al [18] and Havranek et al [19], we employed the SUTSE model to forecast the daily peak and electricity demand. The mentioned study model is estimated using the Gibbs sampling approach, which is of the Markov Chain Monte Carlo (MCMC) method together with the Kalman filter approach. Specifically, since the measurements come with some randomness and losses, the Kalman Filter is employed to estimate the true states of the system. Nonetheless, the Kalman Filter technique needs an initial variance and measurement. Thus, with the help of the Kalman gain, the mentioned method (Kalman Filter) can estimate the true state of the system. Further, to estimate the variance to be applied, the Markov Chain Monte Carlo (MCMC) method precisely the Gibbs sampler, which uses the Wishart distribution to set its prior, was utilized. After obtaining the states, the Gibbs sampler is further used to estimate the variances for the random parts of the model (SUTSE) by conditioning it on other variance parameters and the estimated states. Details of the aforementioned approaches employed are theoretically discussed as follows.

### 2.1 Seemingly unrelated time series equation (SUTSE) model to forecast the peak and short-term electricity demand

Prior to elaborating on the proposed model for forecasting the park and short-term electricity demand, the current study in the first place generally introduces the state-space model and the Kalman filter approach.

#### 2.1.1 State-space approach

Concerning this extant study, as defined by Durbin and Koopman [20], we formulate the model in a linear Gaussian state space following the general format. Based on this we let $y_{t}$ be a time series vector. The state space form is therefore defined as follows:

(1) $$\begin{array}{c} y_{t}=Z_{t}\alpha_{t}+\varepsilon_{t}\;\varepsilon_{t}∼N\left(0,H_{t}\right)\\ \alpha_{t+1}=T_{t}\alpha_{t}+R_{t}\rho_{t}\;\rho_{t}∼N\left(0,Q_{t}\right) \end{array}$$

Notably, the first expression in equation (1) represents the observation equation whereas the second relation represents the state equation. From specified equation (1) $y_{t}$ is a N×1 vector of observations, $\alpha_{t}$ is an unobserved m×1 vector whereas t=1,…T. Also N is the number of times series. The initial state vector $\alpha_{1}∼N\left(\alpha_{1},P_{1}\right)$ is independent of $\rho_{1},\ldots \rho_{T}$ and $\varepsilon_{1},\ldots ,\varepsilon_{N}$ while $\rho_{t}$ are serially and mutually independent for all t.

#### 2.1.2 Kalman filter

Theoretically, the Kalman filter is a recursive procedure to compute the estimator of the state-space vector at time t based on the information available up to that time according to Harvey [21]. Specifically when the shocks follows the Gaussian distribution, the estimator found will be optimal in terms of the mean square error. The recursive equations of the Kalman filter are therefore expressed as follows;

(2a) $$E\left(\alpha_{T+1}|Y_{T}\right)=\alpha_{T+1}=T_{t}\alpha_{t}+K_{t}v_{t}\text{,}$$

(2b) $$V\left(\alpha_{T+1}|Y_{T}\right)=P_{T+1}=T_{t}P_{t}L_{T}^{\prime }+R_{t}Q_{t}R_{T}^{\prime }$$

(2c) $$v_{t}=y_{T}-E\left(y_{T}|Y_{t-1}\right)=y_{T}-Z_{T}\alpha_{t}$$

(2d) $$K_{t}=T_{t}P_{t}Z_{T}^{\prime }F_{t}^{-1}$$

(2e) $$F_{t}=Z_{T}P_{t}Z_{t}^{\prime }+H_{t}$$

(2f) $$L_{t}=T_{t}-K_{t}Z_{t}$$

From Equation (2a), (2b), (2c), (2d), (2e), (2f) the third expression is the innovation vector, the fourth expression stands for the Kalman gain whereas the fifth expression on the other hand denotes the covariance matrix of the innovations, $Y_{t}=\left\{y_{1},\ldots ,y_{t}\right\}$ and $\nu_{t}∼N\left(0,F_{t}\right)$.

**Fig. 1:**
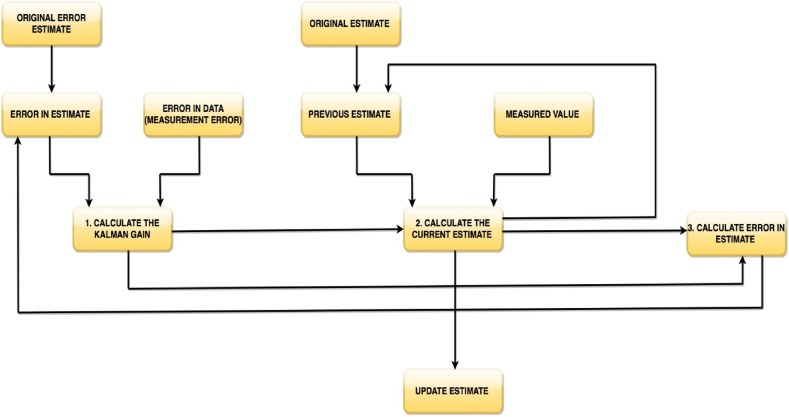
Single measured value Kalman filter.

Further, the Kalman filter is an optimal estimation algorithm. This algorithm is an iterative method that uses a set of equations and sequentially inputs data to estimate the real or true value, velocity, position etc. of an object, with the measured value containing random error, uncertainty or variations. When there are estimated measurements rather than real measurements, or when the observations are considered to have some errors in them, the Kalman filter is mostly employed. A single measure value Kalman Filter is therefore illustrated in Fig. 1 as:

### 2.2 Structural seemingly unrelated times series equation (SUTSE) model

This model is applied on multivariate time series. For the purpose of this study, each of the series is modelled based on the linear growth model. As a result, each series will have state vector that includes the level and slope components, and the system error variance matrix is assumed diagonal for simplicity. This indicates that the growth or evolution of the level and slope is influenced by independent random inputs. Two (2) series, that is m = 2 is considered for this research. The state vector $\theta_{t}=\left(\mu_{1,t},\mu_{2,t},\beta_{1,t},\beta_{2,t}\right)$ has a system equation as;

(3) $$\left[\begin{array}{c} \mu_{1,t}\\ \mu_{2,t}\\ \beta_{1,t}\\ \beta_{2,t} \end{array}\right]=\left[\begin{array}{cccc} 1 & 0 & 1 & 0\\ 0 & 1 & 0 & 1\\ 0 & 0 & 1 & 0\\ 0 & 0 & 0 & 1 \end{array}\right]\left[\begin{array}{c} \mu_{1,t-1}\\ \mu_{2,t-1}\\ \beta_{1,t-1}\\ \beta_{2,t-1} \end{array}\right]+\left[\begin{array}{c} w_{1,t}\\ w_{2,t}\\ w_{3,t}\\ w_{4,t} \end{array}\right]$$

Where ${\left(w_{1,t},w_{2,t},w_{3,t},w_{4,t}\right)}^{\prime }ℵ\left(0,W\right)$ and

(4) $$\left[\begin{array}{c} Y_{1,t}\\ Y_{2,t} \end{array}\right]=\left[\begin{array}{cccc} 1 & 0 & 0 & 0\\ 0 & 1 & 0 & 0 \end{array}\right]\theta_{t}+\left[\begin{array}{c} v_{1,t}\\ v_{2,t} \end{array}\right]$$

represents the observation equation for the bivariate time series with $v_{1,t},v_{t,2}∼N\left(0,V\right)$.

$$\mu_{i,t}\to the\;local\;level$$

$$\beta_{i,t}\to the\;local\;Growth\;for\;i=1,2$$

So, the SUTS model can be written as;

(5) $$y_{t}=\left(F|t⊗I_{m}\right)\theta_{t}+v_{t}v_{t}∼N\left(0,V\right)$$

(6) $$y_{t}=\left(G|t⊗I_{m}\right)\theta_{t}+v_{t}w_{t}∼N\left(0,V\right)$$

Where ⊗ is the Kronecker product and $F_{t}=\left[\begin{array}{cc} 1 & 0 \end{array}\right]$ and $G_{t}=\left[\begin{array}{cc} 1 & 1\\ 0 & 1 \end{array}\right]$.

#### 2.2.1 Local linear trend model

This is a dynamical linear model that incorporates both the local level and the growth in the local level by looking at the trend component as a stochastic term in the model. Due to its process of estimation, it is also referred to as the Local Growth Model. The trend or the slope in this model is a time varying slope which captures the time changing dynamics in terms of changing trends in the data from time to time. In this model, $\theta_{t}={\left(\mu_{t},\beta_{t}\right)}^{\prime }$ becomes the state vector where μ_t_ is mostly explained as the local level and the local growth rate as β_t_. The basic or fundamental idea behind this model is that the level μ_t_ varies or changes linearly over time and there is a likelihood of the growth rate to evolve over time. This method, one is able to capture local trend, thus local increase or decrease in demand across time by looking at the trend component as a stochastic process. The trend is modelled as a random walk similar to the local level which incorporates the previous trend component as a predictor for the current level.

The model is given as

(7a) $$y_{t}=\mu_{t}+V_{t}\;V_{t}∼N\left(0,V\right)$$

(7b) $$\mu_{t}=\mu_{t-1}+\beta_{t-1}+W_{t,1}\;W_{t,1}∼N\left(0,\sigma_{\mu }^{2}\right)$$

(7c) $$\beta_{t}=\beta_{t-1}+W_{t,2}\;W_{t,2}∼N\left(0,\sigma_{\beta }^{2}\right)$$

where $v_{t},w_{t,1},w_{t,2}$ are uncorrelated errors.

### 2.3 Estimation of model parameters

The Gibbs sampling approach was applied in the estimation of the parameters within the SUTS model. Specifically, the Gibbs sampling method is a MCMC method which uses the idea of simulation to estimate the ergodic mean, and the parameters by using the observed data together with the simulated values of all other parameters. Considering the SUTSE system where each series is modelled by a linear growth model. The precision $\Phi_{0}=V^{-1},\Phi_{1}={W_{\mu }}^{-1}\wedge \Phi_{2}={W_{\mu }}^{-1}$ are set as the prior distributions, which follows the Wishart distribution $\Phi_{j}\left(v_{j},S_{j}\right),j=0,1,2\text{.}$ The Wishart hyperparameters can be expressed as

(8) $$v_{0}=\frac{\delta_{0}+m-1}{2}=\frac{\delta_{0}+1}{2}$$

(9) $$v_{j}=\frac{\delta_{0}+p_{j}-1}{2}=\frac{\delta_{j}+1}{2}j=1,2$$

(10) $$S_{0}=\frac{V_{0}}{2}S_{1}=\frac{W_{\mu ,0}}{2}S_{2}=\frac{W_{\beta ,0}}{2}$$

So, if $\delta_{j}>2,j=0,1,2$ then

(11) $$E\left(V\right)=\frac{1}{\delta_{0}-2}E\left(W_{\mu }\right)=\frac{1}{\delta_{1}-2}W_{\mu ,0}E\left(W_{\beta }\right)=\frac{1}{\delta_{1}-2}W_{\beta ,0}$$

Parameters $\delta_{j}$ gives an idea about the uncertainty in the prior. From the full conditional distributions,

(12) $$E\left(V|y_{1:T},W_{\mu },W_{\beta },\theta_{0:t}\right)=\frac{\delta_{0}-2}{\left(\delta |0+T\right)-2E\left(V\right)+\frac{T}{\left(\delta |0+T\right)-2\frac{\sum_{t-1}^{T}\left(y_{t}-F_{t}\theta_{t}\right){\left(y_{t}-F_{t}\theta_{t}\right)}^{\prime }}{T}}}$$

A smaller weight is put on the prior in the update when $\delta_{j}$ is closer to 2. From the joint posterior distribution $\pi \left(\theta_{0:T},\Phi_{0},\Phi_{1},\Phi_{2}\vee y_{1:T}\right)$, the Gibbs sampler generates the state vectors incorporating Kalman filter and the precision $,\Phi_{0},\Phi_{1},\Phi_{2}\text{.}$ Hence, the full conditional distribution of the precisions are given as:

(13) $$\Phi_{0}\vee \ldots W\left(\frac{\delta_{0}+1+T}{2},\frac{1}{2}\left(V_{0}+{SS}_{y}\right)\right)$$

(14) $$\Phi_{1}\vee \ldots W\left(\frac{\delta_{1}+1+T}{2},\frac{1}{2}\left(W_{\mu ,0}+{SS}_{t,1}\right)\right)$$

(15) $$\Phi_{2}\vee \ldots W\left(\frac{\delta_{2}+1+T}{2},\frac{1}{2}\left(W_{\beta ,0}+{SS}_{t,2}\right)\right)$$

Where,

(16) $${SS}_{t,i}=\left(\theta_{t,i}-G_{t,i}\theta_{t,i-1}\right){\left(\theta_{t,i}-G_{t,i}\theta_{t,i-1}\right)}^{\prime }$$

and

(17) $${SS}_{y}=\sum_{t-1}^{T}\left(y_{t}-F_{t}\theta_{t}\right){\left(y_{t}-F_{t}\theta_{t}\right)}^{\prime }$$

#### 2.3.1 Framework of the study

**Fig. 2:**
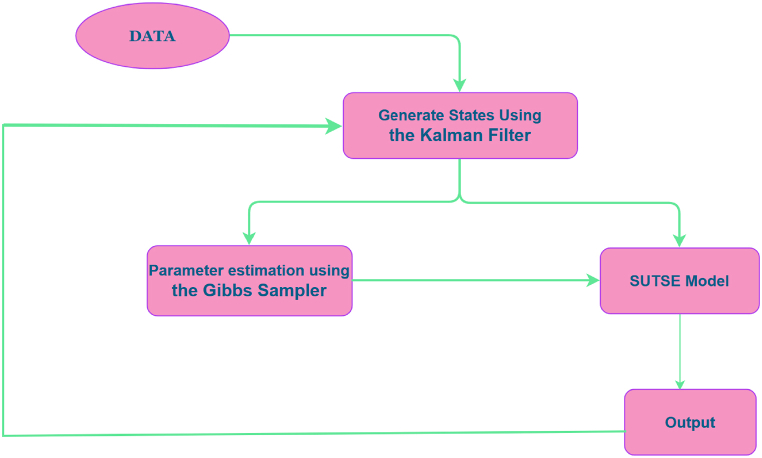
Schematic diagram (study roadmap).

In order to achieve the optimum goal of this extant research, the study follows a proposed framework (roadmap) which is illustrated in Fig. 2. With reference to the mentioned figure, the data or observations are pushed into the Kalman Filter which basically uses the collected data together with its uncertainty to estimate their true states or estimates. The Gibbs sampler also combines the estimate state together with th parameter by setting up a Markov process which goes through the Monte Carlo method to estimate the ergodic means which are actually the parameter estimates for the SUTSE model. From the SUTSE model, the actual estimate is generated.

## 3 Results

### 3.1 Data description

There were 1644 observations each from the daily peak demand data and the daily electricity generation from February 2, 2014 to August 2, 2018 collected from the Ghana Grid Company Limited. The minimum peak demand value recorded over this period was 1228 MW and a maximum value of 2433 MW. The electricity generation data recorded a maximum value of 50832 MWh and a minimum value of 15552 MWh. The mean values of the peak demand and the generation are 1815.88 MW and 35917.48 MWh respectively. The standard deviations in the peak and generation data recorded were 4569.109 MW and 228.9 MWh and respectively. In addition, the Peak demand had a coefficient of variation of 12.6% while the electricity demand had a coefficient of 12.7%. This implies that the variations in both datasets relative to their means are approximately the same. Please see Table 1 for the summarized results.

**Table 1:** Descriptive statistics.

	MIN value	MAX. value	MEAN value	STANDARD DEVIATION	COEFFICIENT OF VARIATION
ELECTRICITY DEMAND	15552	50832	1815.88	228.9	12.6
PEAK DEMAND	1228	2433	35917.48	4569.109	12.7

### 3.2 Peak demand and electricity demand time series plot

**Fig. 3:**
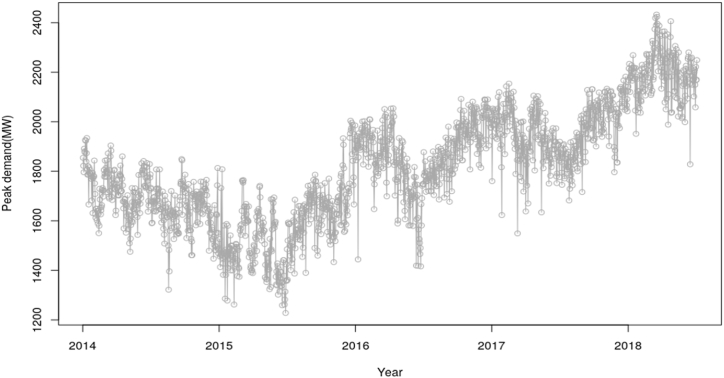
Plot corresponding to the peak demand.

**Fig. 4:**
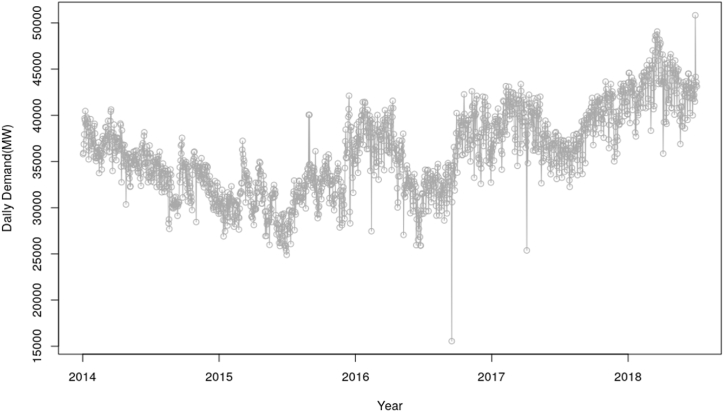
Plot for daily electricity demand.

Fig. 3 shows the peak demand, which demonstrates a high level of noise in the system and also have changing mean and variance across the series, hence likely to be non-stationary too. It also shows a decreasing trend through to the middle of 2015 and started increasing from that point. Further, Fig. 4 shows the daily total energy generation, which also demonstrates a high level of noise in the system. There are also few data points, which are likely to be outliers. The high level of noise in the peak demand data from both Fig. 3, Fig. 4 is likely to make it challenging to accurately forecast future electricity demand. The noise can introduce uncertainty and unpredictability, which tends to affect the accuracy of load forecasting models. This is as a result can lead to potential mismatches between electricity supply and demand, leading to inefficient resources allocation and potential power outages.

### 3.3 Markov Chain Monte Carlo (MCMC) simulation for variance estimation

**Fig. 5:**
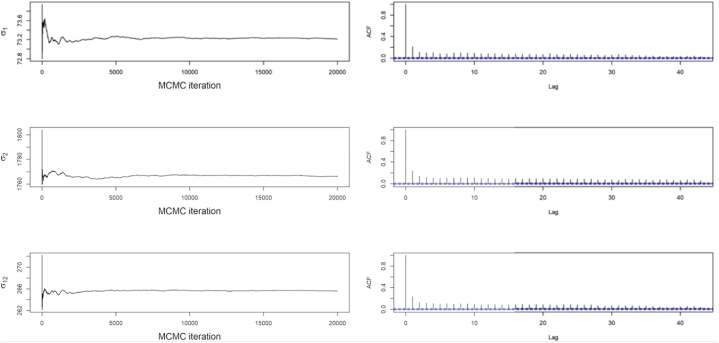
MCMC ergodic means and autocorrelation for the covariance matrix V.

A few diagnostic plots obtained from the MCMC output for the covariance V is shown in Fig. 5. The mentioned figure as well shows the running ergodic means of the parameters and the estimated autocorrelation plots. The converged ergodic mean in Fig. 5 gives variance and covariance. The ACF plots as illustrated in Fig. 5 also demonstrates how weak the correlations between the days grow when the days are far apart.

Since the simulation converges, the posterior means or the ergodic means for the observation covariance matrix were estimated as shown in Eq. (18). Moreover, the deviations in (parenthesis) involved in the Monte Carlo were estimated. Thus

(18) $$E\left(V|y_{1:T}\right)=\left[\begin{array}{l} \begin{array}{l} 5352.923\\ \left(2.5078\right) \end{array}\\ \begin{array}{l} 69829.474\\ \left(52.566\right) \end{array} \end{array}\begin{array}{l} \begin{array}{l} 69829.474\\ \left(52.566\right) \end{array}\\ \begin{array}{l} 3160403.255\\ \left(1679.427\right) \end{array} \end{array}\right]$$

The estimated outcome as illustrated in equation (18), shows that the daily demand and peak demands are positively correlated. This thus implies that fundamentally, there is an expected increase in peak demand in a day if the demand increases and this is depicted in Fig. 3, Fig. 4. Further, the expected peak for a particular day is expected to increase if demand increases because a much higher energy will have to be produced to meet the demand, thereby having a higher peak demand.

**Fig. 6:**
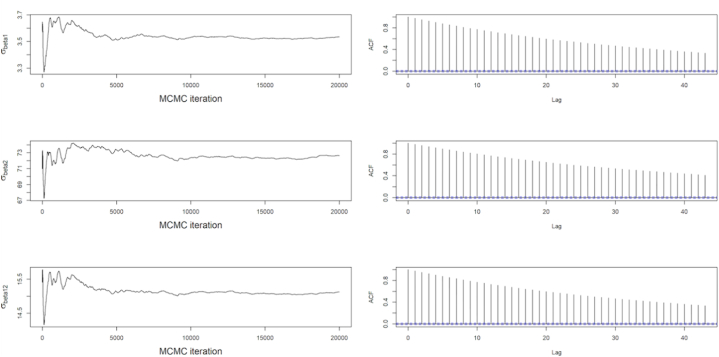
MCMC ergodic means and its corresponding autocorrelation for the covariance matrix *W*_*β*_.

Fig. 6 also on the other hand shows similar diagnostic plots obtained from the MCMC output for covariance matrix in trend component of the system. The mentioned figure additionally shows the running ergodic means of the parameters and the estimated autocorrelation plots. The ACF plots in Fig. 6 demonstrates how weak the correlations between the days grows when the days are far apart from each other yet are still likely to be correlated since the correlation dies out slowly. Hence, demand for day 1 will be highly be correlated with day 2 and also to days which are a little far from day 1. Therefore, higher demand of energy on day 1 will inform a possibly higher demand in day 2 and other closer days.

The posterior means or ergodic means for the trend component in the system together with the Monte Carlo deviations can be estimated from the MCMC output due to convergence of the Monte Carlo process. Thus,

(19) $$E\left(W_{\beta }|y_{1:T}\right)=\left[\begin{array}{l} \begin{array}{l} 12.730\\ \left(0.1657\right) \end{array}\\ \begin{array}{l} 230.371\\ \left(3.211\right) \end{array} \end{array}\begin{array}{l} \begin{array}{l} 230.371\\ \left(3.211\right) \end{array}\\ \begin{array}{l} 5220.107\\ \left(82.454\right) \end{array} \end{array}\right]$$

**Fig. 7:**
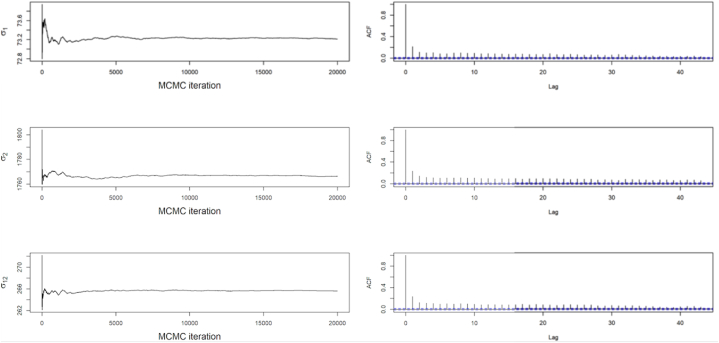
MCMC ergodic means and its corresponding autocorrelation for the covariance matrix *W*_*μ*_

Similarly, compared to Fig. 5, Fig. 6, the ACF from Fig. 7 shows how weak the correlations between the days grows when the days are far apart from each other.

Since Seemingly Unrelated Time Series Equations Model involves integrated random walks, the Ergodic means or posterior means were expected to approach zero as shown in matrix Equation (20).

(20) $$E\left(W_{\mu }|y_{1:T}\right)=\left[\begin{array}{l} \begin{array}{l} 9.982080e-05\\ \left(5.288852e-07\right) \end{array}\\ \begin{array}{l} 5.173045e-07\\ \left(5.288852e-07\right) \end{array} \end{array}\begin{array}{l} \begin{array}{l} 5.173045e-07\\ \left(5.288852e-07\right) \end{array}\\ \begin{array}{l} 9.984811e-05\\ \left(6.293606e-07\right) \end{array} \end{array}\right]$$

### 3.4 Filtering Density and one-step-ahead forecasting plots for the peak and electricity demand

**Fig. 8:**
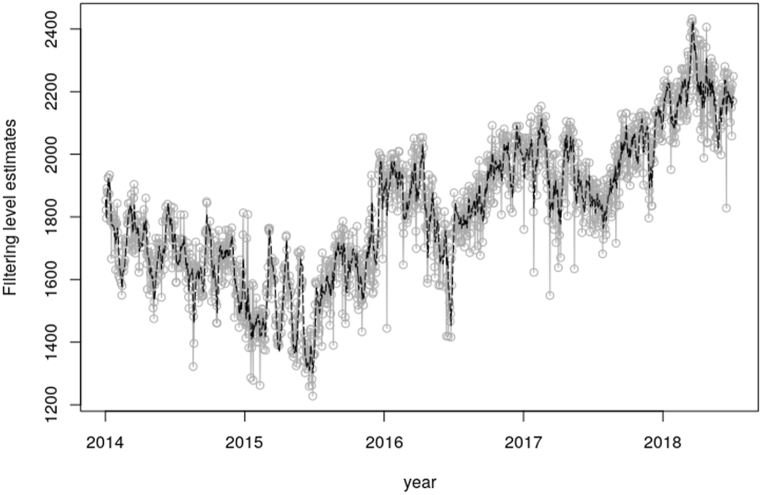
Filtering density for the daily peak demand.

Fig. 8, Fig. 10 shows the estimated means of filtering distribution of the states of the system concerning the peak and electricity demand correspondingly. The estimated mean distributions with respect to the peak and electricity demands indicates the true distribution of the system using the Kalman Gain to help generate a one-step-ahead estimate. The aforementioned Fig. 8, Fig. 10 as well unveils The unobserved states density for the daily total generation resulting from the filtering process. The filtered states as illustrated in the aforesaid figures is the real-time estimation of the underlying load demand states, providing up-to-date information on the current load conditions. This information is vital for short-term load forecasting and load balancing in electricity systems. It does that by capturing the time-varying nature of load demand, allowing for dynamic load modeling. The estimated filtered states from the Kalman filter serve as inputs to adaptive load forecasting models. By continuously updating the state estimates and incorporating them into forecasting algorithms, in this case the SUTSE model, the Kalman filter facilitates adaptive load forecasting, allowing for dynamic adjustments and improvements in forecast accuracy.

**Fig. 9:**
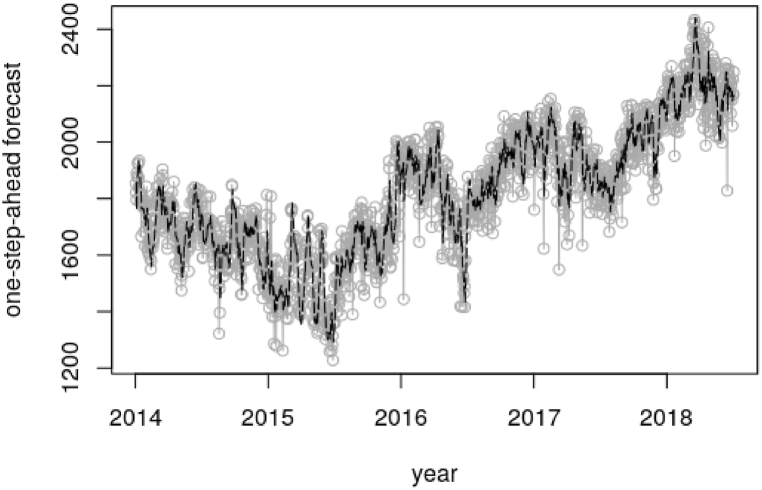
One-step-ahead forecast for daily peak demand.

On the other hand, Fig. 9, Fig. 11 further shows the one-step-ahead predictive distribution for the observations. The one-step-ahead forecasting involves predicting the future values of the peak and electricity demand based on the current state estimates which is provided by the filtered states. The forecasting is done by using the SUTSE model and incorporating the uncertainties associated with the state estimates. The MAPE value from the mentioned Figures involved in this forecasting was 0.03696, which signifies 3.696% error in the forecast and 0.04235, which represents 4.235% error in the forecast respectively. The outlined MAPE estimated values from the aforesaid Fig. 9, Fig. 11 basically gives the indication that the SUTSE proposed model fits very well in predicting the daily demand in the day with a prediction accuracy of approximately 96%. electricity demand.

**Fig. 10:**
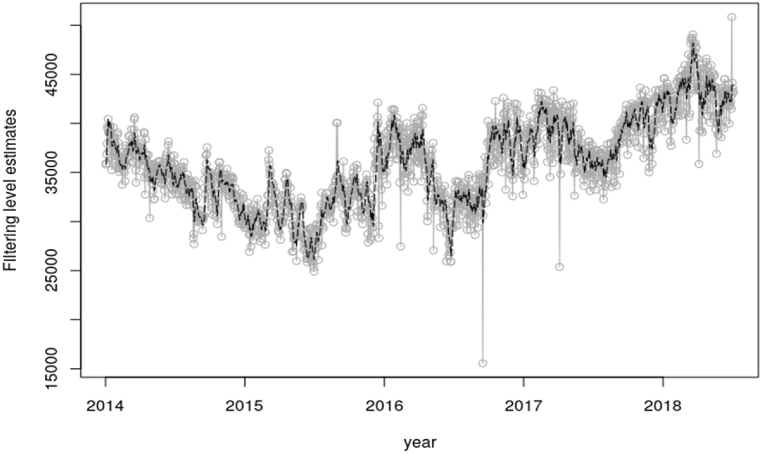
Filtering density for the daily total generation.

**Fig. 11:**
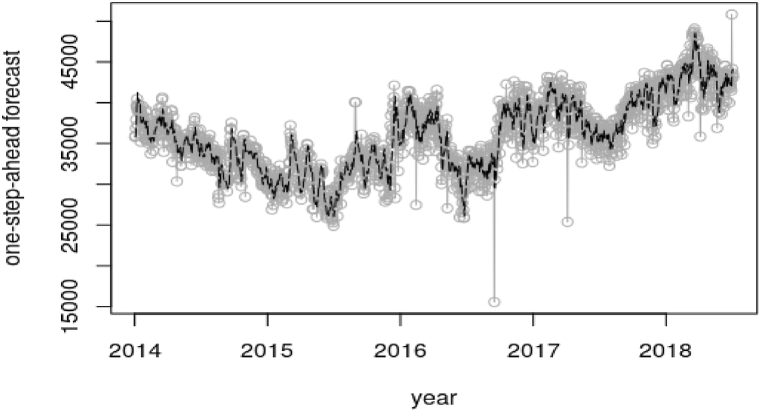
One-step-ahead forecast for the daily electricity demand.

### 3.5 Predicting and forecasting peak demand and electricity demand

Table 2 shows a week forecast from the 28th July to the 2nd August 2018 being compared to the actuals recorded over these same period. Generally, from the accuracy recorded, it is expected that the predicted values for the model with MCMC will be closer to the observed data which is seen in this output. It can be observed that the predictions in the second model (SUTSE model without MCMC) had the first value very close to the observed value as compare to the first model (SUTSE model with MCMC). It can be observed that the forecast values kept increasing in the UTSE model without MCMC kept increasing which does not depict the dynamics in the actuals recorded. Over time, it was also observed that the SUTSE model with MCMC subsequently predicted more accurate as compared to the other model. According to the SUTSE model with MCMC, there is a possible decrease in the daily energy over time.

**Table 2:** Peak and electricity demand prediction.

DATE	Observed Peak (MW)	Observed Demand (MWH)	Peak Forecast values from SUTSE with MCMC model	Demand Forecast values from SUTSE with MCMC model	Peak Forecast values from SUTSE model	Forecast values from SUTSE model
28th July 2018	2209	42808	2168.56	42063.97	2209.18	42519.86
29th July 2018	2149	41491	2166.80	42009.62	2224.58	42622.63
30th July 2018	2058	50832	2165.05	41955.26	2239.98	42725.41
31st July 2018	2222	44161	2163.30	41900.91	2255.38	42828.19
1st July 2018	2168	43294	2161.54	41846.55	2270.78	42930.97
2nd August 2018	2170	43579	2159.79	41792.20	2286.18	43033.75

Further, the outcomes outlined in Table 3 represents the forecasted results pertaining to peak demand and electricity demand for 3rd August. Since the study's interest is based on the short-term forecasting, outlined forecast outcomes infers that, the Ghana Grid company should expect energy demand of about 44090 MW on the 3rd August with the highest energy to supplied (Peak demand) over the day being around 2187 MW.

**Table 3:** Forecast of peak and electricity demand.

Forecast type	Demand for 3rd August 2018
Peak demand	218 MW
Electricity demand	44090 MW

Note: MW represents megawatts.

### 3.6 Model comparison in terms of forecasting performance

Table 4 shows the Mean Absolute Percentage Error (MAPE) which measures the accuracy of the forecast values. The table reveals two methods which are basically the Seemingly Unrelated Time Series Equations (SUTSE) model with and without the Markov Chain Monte Carlo Method (MCMC) for the variance estimation. The SUTSE model which uses the actual variances of the data have errors of 4.13% and 4.51% for the Peak and Daily Energy forecast respectively. This implies that there is about 95.9% accuracy in the forecast of the peak demand and also about 95.5% accuracy in predicting the energy demand. The SUTSE with the MCMC method had recorded an accuracy of about 96% and 95.8% for Peak demand and daily energy respectively. Obviously, the SUTSE which implements the MCMC, specifically the Gibbs sampling for the random part of the model outperforms the other model and hence expected to predict with less error.

**Table 4:** MAPE of peak and electricity demand forecast.

	Peak Forecast values from SUTSE with MCMC (model)	Demand Forecast values from SUTSE with MCMC (model)	Peak Forecast values from SUTSE	Forecast values from SUTSE
MAPE (%)	3.97	4.23	4.13	4.51

## 4 Discussion

The main purpose for this study was to forecast the peak demand and total electricity generation or demand. In view of this, the data collected was visualized and a summary of the data was taken to understand the data. There were two series used for the study; namely the daily peak demand data together with the Daily total electricity or energy generation, which is also referred to as electricity demand data. In all, 1644 observations each were used for this study. The minimum peak demand value recorded was 1228 MW while the maximum value recorded was 2433 MW descriptively. Concerning the electricity generation, the lowest generation recorded was 15552 MW whereas the maximum was 50832 MW. The mean peak demand and electricity demand were also 1816 MW and 35917 MW respectively. In addition, the Peak demand had a coefficient of variation of 12.6% while the electricity demand had a coefficient of 12.7%. This implies that the variations in both datasets relative to their means are almost the same.

Considering the analysis conducted on the peak demand and electricity demand time series plot, Fig. 2 demonstrated a decreasing trend through the middle of 2015 and started increasing from the same point. This decreasing trend in Fig. 3 is expected since Ghana experienced the worse power problems between 2014 and mid of 2015. Even though the demands keeps increasing, Ghana experienced lots of power outages due system failures, dam reconstruction, power facility extensions among other major projects. Due to these reasons, there were shedding of energy and hence the demand per day decreased, leading to reduction of the peak demand within that period. These challenges were minimized after the mid of 2015, hence experiencing increase in demand from that period. Decrease in demand or supply tends to minimize or reduce the highest demand to be recorded. There is also an increasing peak demand from the mid of 2017 through 2018. This might be attributed to the expansion of electricity access and facilities in the country.

In addition, Fig. 4 indicated a high level of noise in system. A few data points were also evidenced to be outliers. This is as a result likely to be attributed to power shortage challenges in the country. The mentioned figure as well shows slight decreasing trend through to mid-2015 and started increasing again from the latter part of 2017. The early decreasing trend which also reflected in Fig. 1 can also be attributed to the power problems which led to the power outages, popularly referred to as the “Dumso” in the country. Since there was an implementation concerning the shedding of energy, some parts of the country at a particular point in time experienced power outages and hence reducing the demand for electricity to be generated and therefore reduction in peak demand as well. There is also a possibility that the expansion of facilities and coverage area in 2018 also increased the demand.

Further the outputs concerning Fig. 5, Fig. 6, Fig. 7 demonstrate the convergence of the Monte Carlo method, Gibbs Sampling applied for the estimation of the parameters in the model, specifically the covariances. The simulation based on the Law of Large Numbers guarantees the convergence of the Gibbs Sampling to the Ergodic Means. Fig. 5 captures the dynamics that estimate the ergodic mean which in this graph it the variance covariance matrix for observation equation. Almost all the component of the parameter achieved convergence within 20,000 simulations. The figure also showed the autocorrelation function (ACF) plot which tells the dynamics of the observations as the days elapse. It was observed that the correlation existing between the days kept dying out. It basically affirms the convergence and advises the operator that the days closer to each other kind of have related energy and peak demand as compared to days far apart from each other. It also affirms the idea of the Markov processes, avoiding using far apart day as a contributor to the current day's demand. Hence, operators are likely to supply similar demand as a day before. Fig. 6, Fig. 7 captures similar dynamics as Fig. 5. In Fig. 5, the ACF plot shows how slow the convergence in estimating the trend is. Hence, takes longer period to die out. This also shows the similar trend patterns at the local level of the demand, thus the trend in the day-to-day demand patterns or the energy supplied pattern. Fig. 6 demonstrates an exponential decreasing patterns in the day-to-day correlations and also convergence within 20,000 simulations. This also informs the dynamics between the days as shown by the ACF plot based on the stronger correlations in daily peak and energy demand between closer days.

The adopted model, Seemingly Unrelated Time Series model was able to capture the various aspects of the electricity transmission including the losses involved through the process of electricity production, which plays a major role in this sector. The Forward Filtering Backward Sampling was used in the Gibbs Sampler together with Markov Chain Monte Carlo Process method to estimate the posterior means of the unknown variances with 50,000 simulations. Precisely, the choice of the number of iterations is to be assured of convergence. Thus the larger the number of iterations included, the more closely the distribution of the sample matches the actual desired distribution. With the MCMC method which is a Monte Carlo process uses the idea of sampling from a known distribution to estimate the mean which will be the parameter of the data. According to the law of large numbers, the sampling mean gets closer to the true mean as the sample gets larger. This thus justifies the implementation of the mentioned number of iterations. The ACF plots showed weakness in correlation between days far apart from each other. This as a result suggest that, the demand on day 1 is more correlated to day 2 and gradually grows apart when the days move further apart. Hence, the demand on day 1 can really inform decisions on demand on day 2 better than given information on decision on demand of a day far apart from day 1. This buttresses the fact that today's energy demand depends on yesterday's demand. Due to the convergence of the simulation, it was legit to estimate the posterior mean of the variances from the MCMC process and the deviations involved in the estimation. The covariance for the state matrix showed less deviation in the estimated values but the covariance for the observation equations projected a larger variance between the second series with itself of 3160404.255 and a higher deviation of 1679.427. The nonzero covariance value in the observation covariance matrix showed that the Peak demand and the Electricity demand are correlated.

The forward filtering which is estimated by Kalman filter was used to generate the state distribution, which portrayed the actual state of the system. The one-step-ahead forecast was used to test the performance of the model in estimating the true observations. To confirm this, the Mean Absolute Percentage Error (MAPE) was used to measure the accuracy of the model. An error of about 3.696% was estimated from the peak demand one-step-ahead forecast. With the energy or electricity demand, the MAPE recorded was about 4.235%. The predicted peak demand for the next day, 3rd August 2018 is 2187 MW and that of the electricity demand is 44090 MW.

## 5 Conclusion and recommendation for further studies

In this paper a multivariate SUTSE framework was proposed to forecast the peak and short-term electricity demand. The Seemingly Unrelated Time Series (SUTS) model was adopted for this study due to its ability to measure the losses involved in the electricity production. A week forecast from the 28th July to the 2nd August 2018 was being compared to the actuals recorded over these same period. Over time, it was also observed that the SUTSE model with MCMC subsequently predicted more accurate as compared to the other model. According to the SUTSE model with MCMC, there seem to be a possible decline in the daily energy over time. Additionally, since the study's interest is based on the short-term forecasting, outlined forecast outcomes infers that, the Ghana Grid company should expect energy demand of about 44090 MW on 3rd August with the highest energy to supplied (Peak demand) over the day being around 2187 MW. More importantly, the Seemingly Unrelated Time Series Equations (SUTSE) model with and without the Markov Chain Monte Carlo Method (MCMC) for the variance estimation were compared. The SUTSE model which uses the actual variances of the data had errors of 4.13% and 4.51% for the Peak and Daily Energy forecast respectively. This implies that there is about 95.9% accuracy in the forecast of the peak demand and also about 95.5% accuracy in predicting the energy demand. Furthermore, the model together with the Forward Filtering Backward Sampling (FFBS) Gibbs Sampler method made it possible for forecasting the next day's peak demand and total electricity to be generated. Although this current research has tempted to utilize as many observations as possible and applied efficient statistical approaches in achieving the study's objective, some limitations are worth recommending for future research. Firstly, the SUTS model does not capture the seasonality component in the system. Thus, we recommend future works to incorporate a model, which capture seasonal component. Secondly, the forward filtering of the algorithm used the linear Kalman filter, which might fail if the system is highly nonlinear. Based on this, the extended Kalman filter or the unscented Kalman filter, which is able to capture the nonlinearity, is recommended. Further, the Gibbs sampler is very powerful in estimating posterior distribution of parameters but if there is a new observation made then the sampler needs to go through the whole simulation process again. To resolve this, it is recommended to apply the sequential Monte Carlo Methods.

## Declaration of competing interest

Authors of this manuscript declare no conflict of interest.
